# Developmental origins of obesity and type 2 diabetes: molecular aspects and role of chemicals

**DOI:** 10.1007/s12199-013-0328-8

**Published:** 2013-02-05

**Authors:** Hidekuni Inadera

**Affiliations:** Department of Public Health, Faculty of Medicine, University of Toyama, 2630 Sugitani, Toyama, 930-0194 Japan

**Keywords:** Chemicals, Developmental origins of health and disease, Epigenetics, Obesity, Type 2 diabetes

## Abstract

Obesity is a leading risk factor for impaired glucose tolerance and type 2 diabetes (T2D). Although the cause of the obesity epidemic is multi-factorial and not entirely clear, the recent acceleration in incidence is too rapid to be accounted for only by genetics, the wide availability of calorie-rich foods, and increasingly sedentary lifestyles. Accumulating data suggest that the important causes of the obesity epidemic may be related to developmental and early life environmental conditions. The concept of the developmental origins of health and disease (DOHaD) suggests that adverse influences early in development, particularly during intrauterine life, may result in permanent changes in the physiology and metabolism of the infant, which in turn result in an increased risk of non-communicable diseases in adulthood. For example, undernutrition during pregnancy and rapid postnatal weight gain are associated with obesity and T2D in the adult offspring. Moreover, increasing evidence suggests that early-life exposure to a wide range of chemicals has a significant impact on the causes of metabolic disorders. Although the underlying molecular mechanisms remain to be determined, these factors can affect epigenetic processes, such as DNA methylation, allowing the developmental environment to modulate gene transcription. The objective of this review article was to summarize recent progress in the biomedical implications of the DOHaD concept, focusing on the pathogenesis of obesity and T2D, and to discuss a future direction for preventive strategies from a public health perspective.

## Introduction

Non-communicable diseases (NCDs), such as obesity, type 2 diabetes (T2D), and the metabolic syndrome, have long been believed to stem from factors typifying the way many people live today, which include an overly high-calorie diet and low level of physical activity. The obesity epidemic continues to pose one of the largest worldwide threats to the health of the population of almost every country in the world [1, 2]. The metabolic syndrome, a complex condition linked to obesity that is characterized by a cluster of risk factors, including hypertension, dyslipidemia, and glucose intolerance, is also becoming increasingly prevalent [3].

The reason for the rapid expansion of this epidemic remains incompletely understood. The recent acceleration in incidence is too rapid to be accounted for only by genetics, increases in the caloric density of food, and declines in physical activity. It is now assumed that environmental factors acting early in life, especially during fetal life, have profound effects on the susceptibility to disease later in life. Several studies, both in humans and experimental animals, suggest that altered risk of adult diseases may be linked to the maternal environmental and nutritional status around conception and implantation [4, 5]. The observation that early human development affects the risk of NCDs in later life has been confirmed by epidemiological studies [6–8]. Specifically, low birth weight (LBW) has been associated with increased blood pressure, dyslipidemia, and impaired glucose metabolism during adulthood [9–12].

In this review, the concept of the development origins of health and disease (DOHaD) and its possible underlying molecular mechanisms are summarized, followed by future directions for preventive strategies for obesity and T2D in the public health sector.

## Developmental origins of health and disease

The concept of DOHaD is based on the assumption that environmental factors acting early in life (usually during fetal life) have profound effects on the predisposition to disease later in life. The idea that developmental factors may influence the susceptibility to disease much later in life was given impetus by a series of epidemiological studies by Barker et al. [13–18]. These authors reported that LBW babies who survived infancy and childhood were at increased risk of having risk factors for coronary heart disease in adulthood. LBW is considered to be a marker for an adverse fetal environment and fetal stress. Correction for known risk factors, such as diet, smoking, and exercise, did not have a major impact on the relationships between LBW and subsequent risk of NCDs [19–23]. Relationships have now been described between LBW and many risk factors for coronary heart disease, including hypertension, dyslipidemia, and T2D [24]. LBW has also been associated with increased insulin resistance later in life [10].

Barker et al. [5, 15] proposed the thrifty phenotype hypothesis in an attempt to explain the associations between poor fetal growth and increased risk of obesity and T2D in adult life. Thrifty genotype confers a survival advantage in a poor food environment by reducing glucose uptake and limiting body growth. However, when individuals of this genotype encounter an environment of plentiful food, they are at risk of developing obesity and T2D. This hypothesis can explain the molecular basis of DOHaD. Since this hypothesis was proposed, many studies worldwide have provided the epidemiological evidence for this concept [16, 19–24].

The concept of DOHaD was further supported by the ‘Dutch hunger winter’ studies. The Dutch hunger winter can be regarded as a unique ‘experiment of history’ and shows how maternal undernutrition during specific gestational time windows may affect later health outcome. The winter of 1944–45 in the Netherlands, which was occupied by the Germans in May 1940, is known as the ‘hunger winter.’ Despite the war, nutrition in the Netherlands had generally been adequate up to October 1944. In October 1944, the German authorities blocked all food supplies to the occupied west of the Netherlands. These were restored immediately after liberation on May 5, 1945. Therefore, children exposed to famine in utero during the hunger winter were well nourished in childhood and had accelerated weight gain [25]. The hunger winter cohort was used to examine how maternal undernutrition during specific gestational time windows affects the subsequent life course of offspring who experienced the famine in utero.

The results of the studies of the Dutch hunger winter have given important insights in the causes of DOHaD. A summary of the study outcomes is provided in Table 1. The offspring of women exposed to famine in early gestation, although of normal birth weight, had increased risk of obesity and a threefold increase in the risk of coronary heart disease as adults [26, 27]. The offspring of women exposed to famine in mid- and late gestation were born smaller than unexposed babies and had an increased risk of impaired glucose tolerance as adults [28, 29]. In addition to the studies on the Dutch hunger winter, many other epidemiological studies have indicated that obesity and T2D are highly prevalent in subjects born with LBW [30–34]. The Great Chinese Famine that affected the Chongqing population during 1959–1961 led to overweight females after 50 years [35]. Individuals exposed in utero and infancy to the Nigerian civil war famine were at increased risk of obesity and T2D about 40 years later [36]. Thus, the effects of LBW on the development of obesity and T2D in adult life have been proven in different studies.

**Table 1:** Studies of the Dutch hunger winter families

Subjects	Major findings	References
Obesity and type 2 diabetes		
Age 19 years	Obesity	Ravelli et al. [6]
Age 50 years	2-h glucose levels were elevated after a glucose load	Ravelli et al. [28]
	Increase in body mass index and waist circumference in women	Ravelli et al. [26]
Age 58 years	2-h glucose levels were elevated after a glucose load	de Rooij SR et al. [127]
	Impaired insulin secretion after a glucose load	de Rooij SR et al. [128]
	Glucose intolerance was differed by PPAR-gamma 2 genotype	de Rooij SR et al. [129]
	Increased weight and fat deposition in women	Stein et al. [130]
	No association between prenatal famine and metabolic syndrome	de Rooij et al. [131]
Lipid profiles		
Age 50 years	Increased ratio of low-density to high-density lipoproteins	Roseboom et al. [132]
Age 59 years	Increased total cholesterol and triglyceride levels in women	Lumey et al. [133]
Blood pressure		
Age 50 years	No differences were found in systolic or diastolic pressure	Roseboom et al. [134]
	No association with adult blood pressure	Roseboom et al. [135]
Age 59 years	Moderate increase in systolic but not diastolic blood pressure	Stein et al. [136]
	Greater blood pressure increase during stress	Painter et al. [137]
Atherosclerosis and mortality		
Age 50 years	Increased prevalence of coronary heart disease in those exposed to the famine in early gestation	Roseboom et al. [138]
	Higher incidence of coronary artery disease	Painter et al. [139]
Age 57 years	No effect on adult mortality	Painter et al. [140]
	Reduced carotid artery intima media thickness	Painter et al. [141]
Age between 18 and 64 years	Higher overall adult mortality risk in women	van Abeelen et al. [142]
Miscellaneous		
Age 24–48 years	Increased risk for schizophrenia	Susser et al. [143]
Age 50 years	Decreased factor VII concentrations	Roseboom et al. [144]
	Increased prevalence of obstructive airways disease	Lopuhaa et al. [145]
Age 58 years	No difference in cortisol concentrations after dexamethasone suppression test	de Rooij et al. [146]

*PPAR* Peroxisome proliferator-activated receptor

Intriguingly, adverse events during pregnancy not only affect the offspring of that pregnancy but also the next generation. Women who were severely undernourished during the first trimester of pregnancy gave birth to babies who were of normal birth weight, but those babies themselves then went on to give birth to smaller babies in the next generation [37]. In a cohort of North American Indians, poor maternal nutrition was reported to be associated with an increased risk of T2D over several generations [21].

## Mechanisms of the developmental origins of metabolic diseases

Although our understanding of the molecular mechanisms underlying the effects of fetal undernutrition and LBW on the development of NCDs later in life is far from complete, possible mechanisms have been proposed. Generally, organisms possess an evolved ability to respond to external signals by adjusting their phenotype during development to match their environment. Thus, poor nutrition of the pregnant mother may signal to the fetus that nutrients are scarce in the postnatal environment. Therefore, a fetus that is exposed to signals it interprets as reflecting nutrient deficiency or maternal stress will adapt its metabolic trajectory to suit an environment of limited energy availability. When the postnatal environment then fails to match the experienced prenatal environment, maladaptation occurs, resulting in onset of obesity and T2D [38]. Indeed, it has been shown that those who were most likely to develop T2D in adult life had LBW and underwent rapid postnatal weight gain [39]. In humans, the combination of LBW and rapid childhood growth has been associated with later insulin resistance [38, 39]. Recent animal studies have confirmed that it is mainly the discrepancy between the pre- and postnatal environments that affects adult onset diseases, rather than gestational undernutrition itself [40, 41]. The process whereby a stimulus or insult during a sensitive or critical period has irreversible long-term effects on development is often referred to as ‘metabolic programming’ [42].

The stress response may be involved in the pathogenesis of DOHaD via the regulation of the hypothalamic–pituitary–adrenal (HPA) axis, which has potent effects on metabolism and vasculature [43, 44]. Changes in the HPA axis have been proposed as a mechanism behind the epidemiological link between LBW and later increased blood pressure [45]. Offspring of rat dams given dexamethasone during pregnancy have reduced birth weight and increased blood pressure and glucose intolerance in adulthood [46, 47]. Intrauterine stress is associated with insulin resistance and accompanying dysregulation of the HPA axis, with chronically excessive adrenal glucocorticoid secretion and increased stress responses [48]. Mechanistic analysis has shown that intrauterine glucocorticoid exposure leads to reduced numbers of glucocorticoid receptors in the hypothalamus, resulting in impaired negative feedback and hence long-term upregulation of the HPA axis after birth [49]. This, in turn, may contribute to increased blood pressure and glucose intolerance. In humans, exposure to antenatal betamethasone caused signs of insulin resistance in adult offspring at 30 years of age [50]. Thus, increased maternal corticosteroid levels as a result of stress induced by reduced nutrient availability induce hypertension in the offspring [51]. A recent study reported that maternal iron restriction, independent of maternal macronutrient or caloric intake, also works as a fetal stressor that programs metabolic and circulatory functions in the offspring [52].

Animal studies are used to clarify the underlying mechanisms of clinical observations in more detail. Fetal nutrition is a key regulator of fetal growth and thus an obvious candidate as an influence on programming [53]. In fact, rats living in a postnatal obesogenic environment have very different physiological responses depending on whether they were born to undernourished or well-fed mothers, with the former becoming more obese, more hypertensive, and more hyperphagic than the latter [54]. Imbalance of protein and carbohydrate intake during pregnancy has been associated with reduced birth weight and increased blood pressure in the offspring [55]. Many studies have shown that prenatal protein restriction results in LBW and programs hypertension in experimental animals [56–58]. Embryos collected from mothers fed a low-protein diet have reduced cell numbers [59]. In particular, a strong correlation has been found between nephron number and birth weight [60], and a reduction in nephron number is associated with increased blood pressure [61]. Exposure of rats to a low-protein diet in utero also decreases β-cell proliferation, islet size, and islet vascularization [62]. Subsequent accelerated growth leads to an excessive metabolic demand on this limited cell mass.

Transcriptome-wide analysis using the Affymetrix Mouse 430A_2.0 array (Affymetrix, Santa Clara, CA) showed that feeding mice a protein-restricted diet between gestational days 10.5 and 17.5 altered the expression of 235 genes in the placenta [63]. In the adult male offspring of dams fed a protein-restricted diet, 311 genes in the liver differed significantly from those in the offspring of control dams, as determined by the Agilent 014879 whole rat genome array (Agilent Technologies, Santa Clara, CA) [64]. Thus, an altered maternal diet during pregnancy induces persistent changes in the transcriptome. Whatever the mechanisms, these events may occur in humans if undernutrition in utero is followed by an abundant postnatal diet, such in small babies born into a food-rich Western society.

## Role of epigenetics

The induction of altered phenotypes during development in response to environmental stimuli involves epigenetic changes. Epigenetic regulation has been defined as “heritable changes in gene function that occur without changes in the nucleotide sequence” [65]. Epigenetic factors include DNA methylations, histone modifications, and microRNAs. Epigenetic changes, in particular in DNA methylation, provide a ‘memory’ of developmental plastic responses to the early environment and are central to the generation of phenotypes and their stability throughout the life course. Methylation at the 5′ position of cytosine in DNA within a CpG dinucleotide (the p denotes the intervening phosphate group) is a common modification in mammalian genomes and constitutes a stable epigenetic mark that is transmitted through DNA replication and cell division [66]. CpG dinucleotides are not randomly distributed throughout the genome but are clustered at the 5′ ends of genes/promoters in regions known as CpG islands. Hypermethylation of these CpG islands is generally associated with transcriptional repression, while hypomethylation of CpG islands is generally associated with transcriptional activation.

Data from animal models suggest that epigenetic processes are an important link between the early life environment and altered metabolism and body composition in the adult offspring [67, 68]. A growing body of literature has reported a role for epigenetic factors in the complex interplay between genes and the environment [4, 69–71]. Epigenetic changes may explain how an altered maternal diet during pregnancy, such as a protein restricted diet, induces persistent changes in the transcriptome.

Environmental developmental influences, such as the maternal diet or chemical exposure, can affect the offspring phenotype via epigenetic effects [72, 73]. A diet that is poor or enriched in methyl donors and cofactors of DNA methylation, especially during fetal growth and development, may influence the epigenotype. For example, a methyl-rich maternal diet during gestation was found to alter the body composition of the offspring in agouti mice, which was accompanied by epigenetic changes in metabolic control genes [74]. Thus, environmentally induced epigenetic modifications alter gene expression and may increase the offspring’s susceptibility for later disease [75, 76].

It has been shown in rodent models that unbalanced maternal diets during pregnancy induce changes in DNA methylation and covalent histone modifications in the 5′ regulatory regions of specific non-imprinted genes, affecting the offspring’s later body composition and metabolic phenotype [77, 78]. For example, the offspring of rat dams fed a protein-restricted diet had lower levels of CpG methylation and greater expression of the peroxisome proliferators-activated receptor (PPAR) α and glucocorticoid receptor (GR) genes in the liver than control animals [77]. Changes in the epigenetic regulation of these genes may result in alterations in the activity of pathways controlled by their target genes, such as phosphoenolpyruvate carboxykinase and acyl-CoA oxidase, which subsequently affects lipid and carbohydrate metabolism. Hypomethylation of the hepatic PPARα and GR promoters has been reported in both F1 and F2 offspring of F0 rats fed a protein-restricted diet during pregnancy without further nutritional challenge to the F1 generation, indicating that changes in the epigenome that occur during development may be passed on to subsequent generations [68].

Intrauterine growth restriction has been associated with progressive epigenetic silencing of *Pdx1*, a pancreatic and duodenal homeobox 1 transcription factor critical for β-cell development, resulting in impaired β-cell function and T2D in the adult offspring in rats [79]. Lack of methylation of the retrotransposon with a methylation-sensitive promoter was associated with subsequent offspring obesity in mice [80]. In contrast, supplementation of the diets of pregnant animals with methyl donors, such as folic acid, vitamin B12, choline, or betaine, increased DNA methylation of specific genes in the offspring [74, 81–83]. Overall, these results show that dietary modifications can induce altered phenotypes through epigenetic changes in specific genes and that these changes in phenotype can be modulated by nutritional interventions during pregnancy.

As yet there are limited published human data linking maternal nutrition to epigenetic changes in the offspring. Adults who were exposed to famine in utero showed altered DNA methylation in the promoters of several imprinted and non-imprinted genes in white blood cells. Two studies on the Dutch hunger winter families reported the effects of prenatal undernutrition on promoter methylation [84, 85]. Individuals with periconceptual exposure to famine had reduced DNA methylation of the imprinted insulin-like growth factor-2 (*IGF2*) gene, a key factor in human growth and development, as compared with their unexposed, same-sex siblings at 59 years of age [84]. In addition, individuals from Dutch hunger winter families also had lower DNA methylation of the imprinted INS-IGF2 (*INSIGF*) gene, but increased DNA methylation of the guanine nucleotide-binding protein (*GNASAS*), maternally expressed 3 (*MEG3*), interleukin-10 (*IL10*), ATP-binding cassette A1 (*ABCA1*), and leptin (*LEP)* genes in parallel with impaired glucose tolerance compared with their unexposed same-sex siblings [85]. A recent study reported that higher methylation of retinoid X receptor-α chr9 was associated with lower maternal carbohydrate intake in early pregnancy, which is also associated with higher neonatal adiposity [53]. Thus, it is clear that fetal stress can affect the methylation status of several subsets of genes, supporting the hypothesis that associations between early developmental conditions and health outcomes later in life may be mediated by changes in the epigenetic information. However, the differences in methylation status of these genes between the exposed and unexposed individuals were relatively small, although statistically significant. Further studies with clear end points are needed to determine whether measurement of epigenetic marks in early life can be used as biomarkers to identify individuals who have experienced environmental perturbations in development and thus who are more likely to develop obesity and metabolic disease in later adulthood. It is crucial to identify such epigenetic marks that are predictive of a later phenotype so that they can be used as relevant biomarkers for disease prevention.

## Role of chemicals

Because the obesity epidemic coincided with the rapid increase in the use of industrial chemicals, the hypothesis that exposure to chemicals, combined with genetic predisposition and consumption of a high-calorie diet, may be a major contributor to the obesity epidemic was proposed nearly 10 years ago [86, 87]. Since then, a number of studies have specifically addressed the effects of environmental chemical exposure on weight gain. Chemicals that affect human fetal development are generally called endocrine-disrupting chemicals (EDCs). EDCs are compounds that act upon the body’s hormonal systems and include industrial contaminants, plastics, pesticides, and other compounds [88]. EDC contamination is a global problem. Bisphenol A (BPA), the prototypical EDC, is produced in large quantities for use in the production of polycarbonate and epoxy resins. However, when used in food and drink containers, it can ‘leach’ into the contents, resulting in ingestion of BPA with food and drink [89]. As a result, human exposure to BPA is widespread, and BPA has been detected in urine in more than 90 % of all human samples tested [89, 90]. Some EDCs are highly resistant to degradation and remain persistent in the environment. For example, persistent organic pollutants, which include polychlorinated dibenzo-*p*-dioxins, polychlorinated dibenzofurans, and polychlorinated biphenyls (PCBs) can accumulate in the human body, especially in adipose tissue because of their lipophilic nature. Although the production of PCBs was banned by the USA in the 1970s, PCBs remain ubiquitous contaminants in the human population even today because of their stability [91]. Many studies have been conducted to study the link between chemical exposure and metabolic disorders. The findings of studies on the effects of environmental chemicals on obesity and T2D are summarized in Table 2.

**Table 2:** Studies on environmental chemicals on obesity and type 2 diabetes

Subjects	Chemicals^a^	Major findings	References
Human studies			
Adults in southern Taiwan	Arsenic	High prevalence of T2D	Lai et al. [147]
Air Force veterans	TCDD	Increased prevalence of T2D	Henriksen et al. [148]
Air Force veterans	TCDD	Serum level was correlated with incidence of T2D	Longnecker et al. [149]
Pubertal boys	DDE	Increased body weight	Gladen et al. [150]
Adults	POPs	Correlated with the prevalence of T2D	Lee et al. [100]
Adult women	PCB	Increased incidence of T2D	Vasiliu et al. [102]
Mexican Americans	*p*, *p*′-DDT	Increased prevalence of T2D	Cox et al. [151]
Adult native Americans	HCB	Serum level was positively correlated with incidence of T2D	Codru et al. [152]
U.S. population	Organochlorine pesticides	Positively associated with metabolic syndrome	Lee et al. [153]
Children aged 6 years	HCB	Increased BMI (body mass index) and body weight	Smink et al. [97]
Yucheng poisoning women	PCBs	Increased prevalence of T2D	Wang et al. [95]
Residents in Cd-contaminated area	Cd	Correlated with diabetic nephropathy	Hanswell-Elkins et al. [154]
Air Force veterans	TCDD	Increased prevalence of T2D	Michalek et al. [155]
Adult female offspring	DDE	Increased weight and BMI	Karmaus et al. [98]
Women aged 50–59 years	*p*, *p*′-DDE	Increased prevalence of T2D	Rignell-Hydbom et al. [156]
Workers	PFOS	Increased prevalence of T2D	Lundin et al. [157]
Children aged 3 years	DDE and PCBs	Intrauterine exposure was associated with BMI	Verhulst et al. [103]
Great Lakes sport fish consumers	DDE	Increased incidence of T2D	Turyk et al. [158]
Women in southern Spain	Nonylphenol	Positively associated with BMI	Lopez-Espinosa et al. [159]
Adults of eastern Slovakia	PCBs	Increased prevalence of T2D	Ukropec et al. [160]
Koreans	Organochlorine pesticides	Increased prevalence of T2D	Son et al. [161]
Adults	PCBs	Increased prevalence of T2D	Lee et al. [162]
Koreans	Heptachlor epoxide	Positively associated with metabolic syndrome	Park et al. [163]
Children at 14 months	DDE	Elevated BMI	Mendez et al. [164]
Adults aged 18–74	BPA	Higher exposure was associated with general and central obesity	Carwile et al. [165]
Women aged 20 years	PFOA	Maternal PFOA concentrations were positively associated with BMI	Halldorsson et al. [104]
Adult populations of Catalonia	PCBs and HCBs	Positively associated with diabetes and prediabetes	Gasull et al. [166]
Animal studies			
Rats	Cd	Neonatal exposure increased diabetic prevalence	Merali et al. [167]
Female mice	BPA	Perinatal exposure led to obesity	Howdeshell et al. [107]
Female rats	BPA	Perinatal exposure led to obesity	Rubin et al. [168]
Rats	BPA	Perinatal exposure led to impaired glucose tolerance	Wei et al. [109]
Female ewe lambs	Octylphenols	Gestational exposure led to obesity	Wright et al. [112]
Mice	DES	Perinatal exposure led to obesity	Newbold et al. [106]
Mice	TBT	In utero exposure led to obesity	Grun et al. [117]
Female mice	BPA	Perinatal and postnatal exposure led to obesity	Miyawaki et al. [169]
Female mice	PFOS	Work as developmental obesogen	Hines et al. [170]
Female rats	BPA	Gestational exposure led to obesity	Somm et al. [108]
Mice	TBT	In utero exposure led to multipotent stem cells to become adipocytes	Kirchner et al. [118]

*TD2* Type 2 diabetes,* BMI* body mass index

^a^ *TCDD* 2, 3, 7, 8-Tetrachlorodibenzo-p-dioxin, *DDE* dichlorodiphenyl-dichloroethylene, *POPs* persistent organic pollutants, *PCB* polychlorinated biphenyl, *p*, *p*′*-DDT* dichlorodiphenyltrichloroethane, *HCB* hexachlorobenzene, *Cd* cadmium, *PFOS* perfluorooctanesulfonic acid, *BPA* bisphenol A, *PFOA* perfluorooctanoate, *DES* diethylstilbestrol, *TBT* tributyltin, *PFOS* perfluorooctanoic acid

Epidemiological studies on maternal smoking have indicated that the adjusted odds ratio for obesity is between 1.5- and 2.0-fold greater if children were exposed during, but not before or after, pregnancy [92–94], suggesting that there are critical periods during embryogenesis when the embryo is the most sensitive to exposure to xenobiotics. These studies also indicated that chemical toxicants, such as nicotine, can contribute to the etiology of later obesity. In humans, there is epidemiological evidence for the association between developmental exposure to chemicals and metabolic disorders later in life [95]. It has been demonstrated that chemicals cross the placenta and directly affect the fetus [96]. Maternal exposure to chemical substances during pregnancy has been associated with an increased body mass index (BMI) in the offspring [97–99]. Several studies have indicated that organochlorine exposure may be associated with the development of T2D [97, 98, 100–103]. For example, the results of a Spanish cohort study showed that prenatal exposure to the organochlorine hexachlorobenzene was associated with increased BMI at age 6 years [97]. Prenatal exposure to dichlorodiphenyl-dichloroethylene (DDE) was significantly associated with increased weight and BMI in adult female offspring [98]. DDE in cord blood was associated with increased BMI in young children, and this effect was exacerbated by maternal smoking [103]. A recent prospective study reported that in utero exposure to perfluorooctanoate was positively associated with risk of overweight at age 20 years in female but not in male offspring [104]. Collectively, these results support the hypothesis that the fetus is vulnerable to exposure to environmental chemicals, resulting in an increased risk of excessive body weight gain and susceptibility to T2D later in life. Moreover, gender and the postnatal environment in combination with prenatal chemical exposure can modify the onset, as well as the progression and outcome of the disease.

In experimental cell cultures and animal studies, a variety of chemicals have been shown to act as adipogenesis or obesity-inducing agents. A number of chemicals are known to promote obesity by increasing the number of adipocytes or the storage of fat into existing adipocytes. The organochlorine compound 1, 1, 1-trichloro-2, 2-bis(*p*-chlorophenyl)-ethane (*p*, *p*′-DDT) induces a concentration-dependent increase in in vitro 3T3-L1 adipocyte differentiation [105]. In mouse studies, treatment with the synthetic estrogen diethylstilbestrol (DES) on days 1–5 of neonatal life at 0.001 mg/day increased body weight and the percentage of body fat [106]. Treatment of pregnant mice with xenoestrogen BPA resulted in increased body weight in the female offspring on postnatal day 22 compared with unexposed controls [107]. Recent studies have indicated that perinatal exposure to approximately 70 μg/kg/day BPA via drinking water alters early adipogenesis and increases body weight in rodent models [108, 109]. Mice perinatally exposed to DES or the phytoestrogen genistein increased weight after puberty [106, 110, 111]. Wright et al. [112] reported that exposure to octylphenol, another xenoestrogen, during fetal and postnatal life in female lambs led to increased weight at puberty. During development, estrogen induces an increase in adipocyte numbers and affects adipocyte function [113]. Overall, these results show that exposure to organochlorine compounds and/or xenoestrogens during sensitive windows of development may have obesogenic effects, and may lead to permanent changes in the metabolic pathways that regulate body weight.

Other chemicals that can work as potential obesogens are the organotins. Organotins represent a class of persistent organic pollutants that may reach harmful levels in exposed populations [114]. In culture systems, organotins induce adipocyte differentiation [115, 116]. Tributyltin is also known to induce adipogenesis in vivo. Mice treated prenatally with tributyltin are born with more stored fat than controls [117]. Multipotent stromal cells harvested from white adipose tissue at 8 weeks of age in mice prenatally exposed to tributyltin had high numbers of preadipocytes and cells preprogrammed to prefer the adipogenic fate, an effect that will likely lead to an increase in adipose mass over time [118]. Organotins work via direct activation of the PPARγ–retinoid X receptor (RXR) heterodimer [116]. Activation of the PPARγ–RXR heterodimer has been found to favor lipid biosynthesis and storage, and induces adipocyte differentiation. Interestingly, a recent report indicated that tributyltin can work as a xenoestrogen via estrogen receptors in vivo [119]. Among commonly used phthalates as plasticizers, monoethyl-hexyl-phthalate can work as an activator for PPARγ [120].

The mechanisms of action of these chemicals are diverse and probably involve epigenetic molecular changes, including DNA methylation and histone modifications [121]. Environmental compounds may induce the establishment of specific epigenetic patterns during key developmental periods that influence phenotypic variation, which in some cases lead to disease states [122, 123]. Indeed, maternal exposure to BPA decreased DNA methylation in the retrotransposon upstream of the agouti gene in mice and shifted coat color distribution in the offspring by stably altering the epigenome [74]. Whatever the mechanism, complex events, including exposure to obesogenic or diabetogenic chemicals during development, may be contributing to the obesity and T2D epidemics. Clarifying the mechanisms involved in weight homeostasis is a novel target in the study of abnormal programming induced by environmental chemicals, which should be re-named ‘metabolic disrupting chemicals’ [124].

## Future perspectives

**Fig. 1:**
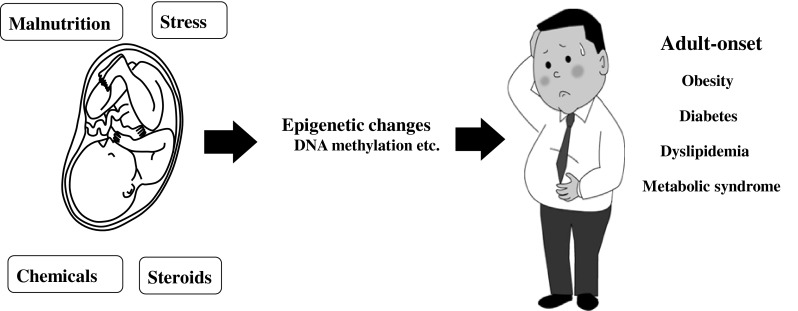
Influences during critical fetal periods may cause the adult onset of non-communicable diseases, such as obesity and type 2 diabetes

We are in the midst of a global epidemic of obesity with substantial negative health and socio-economic consequences. There is now growing evidence that developmental influences have lifelong effects on metabolic function (Fig. 1). Perturbations of the developmental milieu can have a profound impact on the onset and incidence of obesity and T2D. Phenotypic outcomes with long-term consequences involve the interplay between environmental, developmental, and genetic influences. The pathogenesis of obesity and T2D resides in a mixture of genetic and environmental factors. It is therefore probable that not only maternal nutrition and stress, but also maternal size, parity, and maternal age can affect the offspring phenotype and/or epigenetic states. Moreover, possible interplay among prenatal exposure and the postnatal environmental factors, such as nutrition, stress, chemical exposure, and aging, can affect disease outcome. Further studies are merited to clarify the interactions that mediate metabolic outcomes.

Transient environmental conditions during human gestation can be recorded as persistent changes in the epigenome. Epigenetic changes, in particular those in DNA methylation, are central to the generation of novel phenotypes and their stability throughout the life course. In the future, epigenetic research may have substantial benefits for public health because specific components of the epigenetic state at birth may predict later obesity and T2D. Researchers need to identify such effective epigenetic biomarkers which can be measured at early stage of life. These epigenetic marks may be important biomarkers, which not only indicate responses to prenatal challenges, but also predict later risk for obesity and T2D. Current studies are limited to analyzing specific candidate loci, and the complete epigenome has yet to be explored. New technologies, such as methods for rapid sequencing for differentially methylated regions of the genome, need to be developed before large-scale epidemiological studies can be conducted. It is likely that comprehensive studies of the epigenome will be helpful to shed light on gene–environment interactions in the pathogenesis of obesity and T2D.

Accumulating data indicate that low doses of environmental chemicals have adverse effects on human health [125]. These days, individuals are exposed to a mixture of EDCs. Indeed, dozens of environmental chemicals are detected in human tissues and fluids [126]. However, very little is known about how these chemicals act in combination. So far, almost all epidemiological studies have looked for the associations between metabolic disorders and the exposure to a single EDC at a single time, rather than to the whole mixture of toxicants to which human are exposed. These mixtures are likely to have unexpected and unpredictable effects. Further prospective studies with clear end points are required to determine whether exposure to mixtures of EDCs in early life can influence the development of obesity and T2D.

NCDs are preventable, but new initiatives are needed to institute prevention. If the risk of many common diseases of adulthood in our communities is largely determined before birth, adult lifestyle interventions will only reduce the risk transiently or to a small degree because they occur too late. Thus, from a public health perspective, it is important to determine whether interventions after the neonatal period can reverse the adverse effects of unbalanced prenatal nutrition. If the effects of adult lifestyle interventions are limited, maximum effect will be gained from timely interventions in early life. Improved nutrition will not only benefit the present population but may also reduce disease in future generations. If so, more care should be given to the consumption of a healthy diet during pregnancy and improved fetal nutrient availability, which may lead to a more normal birth weight and early life growth, thereby reducing the risk for programmed metabolic disease. Clear evidence and good communication of this evidence may lead to policy responses that will open the possibility of nutritional or pharmacological interventions to combat the rapid rise in obesity and T2D.

## Abbreviations

BMI: Body mass index
BPA: Bisphenol A
DDE: Dichlorodiphenyl-dichloroethylene
*p*, *p*′-DDT: 1,1,1-trichloro-2, 2-bis(*p*-chlorophenyl)-ethane
DES: Diethylstilbestrol
DOHaD: Developmental origins of health and disease
EDC: Endocrine-disrupting chemical
GR: Glucocorticoid receptor
HPA: Hypothalamic–pituitary–adrenal
LBW: Low birth weight
NCDs: Non-communicable diseases
PCB: Polychlorinated biphenyls
PPAR: Peroxisome proliferator-activated receptor
T2D: Type 2 diabetes
